# Dental Exostosis

**Published:** 1846-09

**Authors:** 


*Dental Exostosis.*—The most remarkable example of dental exostosis which we have ever seen, was presented by Dr. James Robinson, of London, to the Museum of the Baltimore College of Dental Surgery. The exostosis is situated upon the posterior part of the neck and root of an inferior dens sapientiæ. It forms a protuberance nearly twice as large as the tooth and projects back from it about seven-eighths of an inch, and the circumference of the distal extremity is more than double that of its neck.—*Balt. Ed.*

